# 2-(2*H*-Benzotriazol-2-yl)-4-methyl­phenyl diphenyl­phosphinate

**DOI:** 10.1107/S1600536809029870

**Published:** 2009-07-31

**Authors:** Yi-Chang Liu, Chia-Her Lin, Bao-Tsan Ko

**Affiliations:** a Department of Chemistry, Chung Yuan Christian University, Chung-Li 320, Taiwan

## Abstract

In the title mol­ecule, C_25_H_20_N_3_O_2_P, the dihedral angle between the mean planes of the benzotriazol ring system and the *N*-bonded benzene ring is 45.8 (2)°. All but one of the angles at the P atom show slight distortions from an ideal tetra­hedral geometry.

## Related literature

For background to the use of 2-(2*H*-benzotriazol-2*H*-yl)phenol (BTP-H) derivatives, see: Li *et al.* (2009 ▶); Tsai *et al.* (2009 ▶). For related structures, see: Al-Farhan (1992 ▶); Cheng *et al.* (2007 ▶).
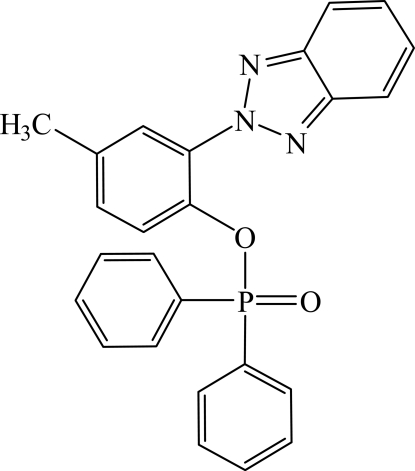


## Experimental

### 

#### Crystal data

C_25_H_20_N_3_O_2_P
*M* *_r_* = 425.41
Orthorhombic, 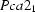

*a* = 12.7691 (7) Å
*b* = 9.4064 (5) Å
*c* = 18.6362 (10) Å
*V* = 2238.4 (2) Å^3^
*Z* = 4
Mo *K*α radiation
μ = 0.15 mm^−1^
*T* = 296 K
0.30 × 0.20 × 0.15 mm

#### Data collection

Bruker SMART-1000 CCD diffractometer
Absorption correction: multi-scan (*SADABS*; Sheldrick, 1996 ▶) *T*_min_ = 0.963, *T*_max_ = 0.974
11920 measured reflections
3684 independent reflections
3343 reflections with *I* > 2σ(*I*)
*R*_int_ = 0.034

#### Refinement

*R*[*F* ^2^ > 2σ(*F* ^2^)] = 0.032
*wR*(*F* ^2^) = 0.086
*S* = 1.05
3684 reflections
280 parameters
1 restraint
H-atom parameters constrained
Δρ_max_ = 0.14 e Å^−3^
Δρ_min_ = −0.20 e Å^−3^
Absolute structure: Flack (1983 ▶), 1412 Friedel pairs
Flack parameter: 0.01 (7)

### 

Data collection: *SMART* (Bruker, 1999 ▶); cell refinement: *SAINT* (Bruker, 1999 ▶); data reduction: *SAINT*; program(s) used to solve structure: *SHELXS97* (Sheldrick, 2008 ▶); program(s) used to refine structure: *SHELXL97* (Sheldrick, 2008 ▶); molecular graphics: *SHELXTL* (Sheldrick, 2008 ▶); software used to prepare material for publication: *SHELXTL*.

## Supplementary Material

Crystal structure: contains datablocks I, global. DOI: 10.1107/S1600536809029870/lh2871sup1.cif

Structure factors: contains datablocks I. DOI: 10.1107/S1600536809029870/lh2871Isup2.hkl

Additional supplementary materials:  crystallographic information; 3D view; checkCIF report

## Figures and Tables

**Table 1:** Selected bond angles (°)

O2—P—O1	115.53 (10)
O2—P—C14	113.48 (8)
O1—P—C14	100.15 (8)
O2—P—C20	112.37 (9)
O1—P—C20	105.04 (8)
C14—P—C20	109.30 (8)

**Fig. 1.**
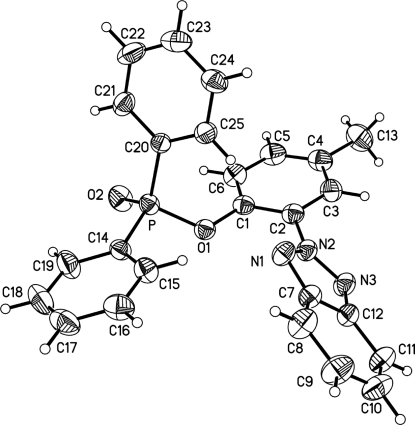
The molecular structure of I with the atom numbering scheme. Displacement ellipsoids are drawn at the 30% probability level.

**Fig. 2.**
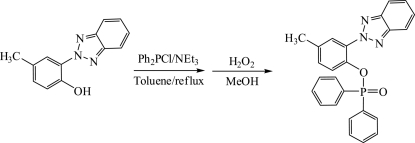
The synthetic procedure of the title compound.

## supplementary crystallographic information

### Comment

2-(2*H*-benzotriazol-2*H*-yl)phenol (BTP-H) derivatives are widely used as ultraviolet (UV) absorbers for the protection of commercially important synthetic plastics and fibers against the UV light in industry. In terms of coordination chemistry, the benzotriazol-phenolate group can provide N, O-bidentate chelation to stabilize the transition metal or main group metal complexes. For instance, our group has successfully synthesized and structural characterized the Pd complex (II) with 4-methyl-2-(2*H*-benzotriazol-2-yl)-phenolate ligands (Tsai *et al.*, 2009). Recently, we also reported the synthesis and crystal structure of an Al(III) complex with the 4-methyl-2-(2*H*-benzotriazol-2-yl)-phenolate ligand (Li *et al.*, 2009). Most recently, Cheng *et al.* (2007) reported some palladium complexes of monodentate phosphinite ligands and these complexes in the presence of Pd(*OAc*)_2_ have been demonstrated effectively to catalyze Suzuki–Miyaura cross-coupling reactions. Therefore, our group is interested in the synthesis and preparation of the phosphinite functionalized benzotriazol-phenolate ligands derived from BTP-H. Here, we report the synthesis and crystal structure of the title compound, (I), a potential ligand for the preparation of metal complexes.

The molecule of (I) is composed of a benzotriazol-phenolate moiety and a diphenylphosphine oxide functionalized group (Fig. 1). The dihedral angle between the planes of the benzotriazole moiety and the benzene ring of the phenoxy group is 45.8 (2)°. The P atom is bonded one O atom of the phosphine oxide, one phenoxy O atom and two C atoms from two phenyl groups, forming a slightly distorted tetrahedral environment. The P-O and P=O bond distances bond distances are similar to those found in the crystal structure of triphenyphosphine oxide (Al-Farhan, 1992).

### Experimental

The title compound I was synthesized by the following procedure (Fig. 2): To a rapidly stirred solution of 4-methyl-2-(2*H*-benzotriazol-2-yl)phenol (2.48 g, 10.0 mmol) in toluene (20 ml),  Ph_2_PCl (1.8 ml, 10.0 mmol) and NEt_3_ (20.0 mmol, 2.02 g) was slowly added. The mixed solution was stirred at 363 K for 18 h. Subsequently, the HNEt_3_Cl salt was filtered and the resulting solution was dried under reduced pressure. The resulting oily product was re-dissolved in MeOH (20 ml) and H_2_O_2_ (1 ml) was added. The final solution was stirred at room temperature for another 1 h and the volatile components  were removed in *vacuo*. The residue was extracted with ethyl acetate (50 ml) and the extract was dried under vacuum to give oily, white solids. Colorless crystals were obtained on cooling the saturated Et_2_O solution at 253 K overnight. ^1^H NMR (CDCl_3_, p.p.m.): δ 7.10–7.97 (17*H*, m, Ar*H*), 2.35 (3*H*, s, C*H*_3_).

### Refinement

The H atoms were placed in idealized positions and constrained to ride on their parent atoms, with C—H = 0.93 and 0.96 Å with *U*_iso_(H) = 1.2 and 1.5*U*_eq_(C).

### Crystal data

**Table:**

C_25_H_20_N_3_O_2_P	*F*(000) = 888
*M**_r_* = 425.41	*D*_x_ = 1.262 Mg m^−^^3^
Orthorhombic, *P**c**a*2_1_	Mo *K*α radiation, λ = 0.71073 Å
Hall symbol: P 2c -2ac	Cell parameters from 6495 reflections
*a* = 12.7691 (7) Å	θ = 2.7–26.0°
*b* = 9.4064 (5) Å	µ = 0.15 mm^−^^1^
*c* = 18.6362 (10) Å	*T* = 296 K
*V* = 2238.4 (2) Å^3^	Block, colourless
*Z* = 4	0.30 × 0.20 × 0.15 mm

### Data collection

**Table:**

Bruker SMART-1000 CCD diffractometer	3684 independent reflections
Radiation source: fine-focus sealed tube	3343 reflections with *I* > 2σ(*I*)
graphite	*R*_int_ = 0.034
ω scans	θ_max_ = 26.0°, θ_min_ = 2.2°
Absorption correction: multi-scan (*SADABS*; Sheldrick, 1996)	*h* = −15→14
*T*_min_ = 0.963, *T*_max_ = 0.974	*k* = −11→11
11920 measured reflections	*l* = −19→22

### Refinement

**Table:**

Refinement on *F*^2^	Secondary atom site location: difference Fourier map
Least-squares matrix: full	Hydrogen site location: inferred from neighbouring sites
*R*[*F*^2^ > 2σ(*F*^2^)] = 0.032	H-atom parameters constrained
*wR*(*F*^2^) = 0.086	*w* = 1/[σ^2^(*F*_o_^2^) + (0.058*P*)^2^] where *P* = (*F*_o_^2^ + 2*F*_c_^2^)/3
*S* = 1.05	(Δ/σ)_max_ = 0.001
3684 reflections	Δρ_max_ = 0.14 e Å^−^^3^
280 parameters	Δρ_min_ = −0.20 e Å^−^^3^
1 restraint	Absolute structure: Flack (1983), 1412 Friedel pairs
Primary atom site location: structure-invariant direct methods	Flack parameter: 0.01 (7)

### Special details

**Table:**

Geometry. All e.s.d.'s (except the e.s.d. in the dihedral angle between two l.s. planes) are estimated using the full covariance matrix. The cell e.s.d.'s are taken into account individually in the estimation of e.s.d.'s in distances, angles and torsion angles; correlations between e.s.d.'s in cell parameters are only used when they are defined by crystal symmetry. An approximate (isotropic) treatment of cell e.s.d.'s is used for estimating e.s.d.'s involving l.s. planes.
Refinement. Refinement of *F*^2^ against ALL reflections. The weighted *R*-factor *wR* and goodness of fit *S* are based on *F*^2^, conventional *R*-factors *R* are based on *F*, with *F* set to zero for negative *F*^2^. The threshold expression of *F*^2^ > σ(*F*^2^) is used only for calculating *R*-factors(gt) *etc*. and is not relevant to the choice of reflections for refinement. *R*-factors based on *F*^2^ are statistically about twice as large as those based on *F*, and *R*- factors based on ALL data will be even larger.

### Fractional atomic coordinates and isotropic or equivalent isotropic displacement parameters (Å^2^)

**Table:**

	*x*	*y*	*z*	*U*_iso_*/*U*_eq_	
P	−0.78562 (3)	−0.27388 (4)	−0.41236 (3)	0.04851 (13)	
O1	−0.74629 (11)	−0.25123 (13)	−0.49386 (7)	0.0523 (3)	
O2	−0.89556 (10)	−0.23744 (14)	−0.39862 (10)	0.0724 (4)	
N1	−0.54417 (13)	−0.30745 (18)	−0.54995 (9)	0.0573 (4)	
N2	−0.58778 (12)	−0.21611 (16)	−0.59589 (8)	0.0510 (3)	
N3	−0.56809 (15)	−0.23649 (17)	−0.66558 (9)	0.0617 (4)	
C1	−0.73324 (14)	−0.11822 (19)	−0.52534 (11)	0.0498 (4)	
C2	−0.65289 (15)	−0.10047 (19)	−0.57421 (10)	0.0536 (4)	
C3	−0.63716 (18)	0.0309 (2)	−0.60682 (12)	0.0641 (5)	
H3B	−0.5823	0.0422	−0.6391	0.077*	
C4	−0.7020 (2)	0.1451 (2)	−0.59187 (13)	0.0675 (6)	
C5	−0.78319 (19)	0.1244 (2)	−0.54483 (14)	0.0710 (6)	
H5A	−0.8283	0.1996	−0.5351	0.085*	
C6	−0.80000 (17)	−0.0052 (2)	−0.51143 (12)	0.0652 (5)	
H6A	−0.8558	−0.0163	−0.4798	0.078*	
C7	−0.49173 (14)	−0.3965 (2)	−0.59453 (10)	0.0543 (4)	
C8	−0.4308 (2)	−0.5185 (3)	−0.57796 (13)	0.0768 (6)	
H8A	−0.4209	−0.5492	−0.5310	0.092*	
C9	−0.3876 (2)	−0.5879 (3)	−0.63507 (16)	0.0861 (7)	
H9A	−0.3471	−0.6683	−0.6265	0.103*	
C10	−0.4020 (2)	−0.5427 (3)	−0.70636 (15)	0.0862 (7)	
H10A	−0.3709	−0.5943	−0.7432	0.103*	
C11	−0.45980 (19)	−0.4263 (3)	−0.72317 (13)	0.0762 (6)	
H11A	−0.4683	−0.3963	−0.7704	0.091*	
C12	−0.50631 (15)	−0.3531 (2)	−0.66534 (11)	0.0561 (4)	
C13	−0.6860 (3)	0.2874 (3)	−0.6283 (2)	0.0984 (9)	
H13A	−0.7377	0.3535	−0.6114	0.148*	
H13B	−0.6929	0.2760	−0.6793	0.148*	
H13C	−0.6173	0.3227	−0.6173	0.148*	
C14	−0.75764 (14)	−0.45832 (18)	−0.40115 (10)	0.0511 (4)	
C15	−0.66460 (14)	−0.5202 (2)	−0.42229 (12)	0.0612 (5)	
H15A	−0.6144	−0.4658	−0.4459	0.073*	
C16	−0.64533 (18)	−0.6626 (2)	−0.40869 (16)	0.0746 (6)	
H16A	−0.5822	−0.7036	−0.4226	0.089*	
C17	−0.7204 (3)	−0.7429 (2)	−0.37445 (17)	0.0840 (8)	
H17A	−0.7086	−0.8391	−0.3663	0.101*	
C18	−0.8118 (3)	−0.6822 (3)	−0.35251 (19)	0.0982 (9)	
H18A	−0.8609	−0.7367	−0.3281	0.118*	
C19	−0.83227 (19)	−0.5408 (3)	−0.36614 (16)	0.0811 (7)	
H19A	−0.8956	−0.5008	−0.3520	0.097*	
C20	−0.69692 (13)	−0.17140 (17)	−0.35845 (9)	0.0460 (4)	
C21	−0.73139 (19)	−0.1249 (2)	−0.29154 (12)	0.0636 (6)	
H21A	−0.7985	−0.1470	−0.2757	0.076*	
C22	−0.6643 (2)	−0.0451 (3)	−0.24858 (12)	0.0791 (7)	
H22A	−0.6865	−0.0153	−0.2035	0.095*	
C23	−0.5668 (2)	−0.0102 (2)	−0.27184 (15)	0.0770 (6)	
H23A	−0.5234	0.0449	−0.2430	0.092*	
C24	−0.53189 (18)	−0.0559 (2)	−0.33777 (13)	0.0677 (5)	
H24A	−0.4653	−0.0310	−0.3535	0.081*	
C25	−0.59558 (14)	−0.1389 (2)	−0.38062 (11)	0.0562 (4)	
H25A	−0.5708	−0.1731	−0.4243	0.067*	

### Atomic displacement parameters (Å^2^)

**Table:**

	*U*^11^	*U*^22^	*U*^33^	*U*^12^	*U*^13^	*U*^23^
P	0.0399 (2)	0.0488 (2)	0.0569 (3)	−0.00062 (15)	0.0029 (2)	−0.0003 (2)
O1	0.0566 (7)	0.0499 (6)	0.0504 (7)	0.0003 (5)	−0.0023 (6)	0.0000 (5)
O2	0.0446 (7)	0.0726 (9)	0.0998 (14)	0.0035 (6)	0.0074 (7)	0.0013 (8)
N1	0.0617 (9)	0.0667 (10)	0.0436 (8)	0.0101 (8)	−0.0017 (7)	0.0031 (7)
N2	0.0576 (9)	0.0532 (8)	0.0424 (8)	0.0008 (6)	−0.0003 (6)	0.0025 (6)
N3	0.0772 (11)	0.0662 (10)	0.0416 (9)	0.0011 (8)	0.0019 (8)	0.0047 (7)
C1	0.0529 (10)	0.0484 (10)	0.0481 (10)	0.0013 (7)	−0.0086 (8)	−0.0012 (8)
C2	0.0621 (10)	0.0524 (10)	0.0462 (10)	0.0023 (8)	−0.0080 (8)	0.0007 (8)
C3	0.0749 (13)	0.0586 (12)	0.0588 (12)	−0.0026 (9)	−0.0006 (9)	0.0065 (9)
C4	0.0864 (14)	0.0510 (11)	0.0650 (14)	0.0023 (9)	−0.0146 (11)	0.0061 (10)
C5	0.0842 (14)	0.0547 (11)	0.0742 (15)	0.0183 (10)	−0.0125 (12)	−0.0007 (10)
C6	0.0687 (12)	0.0643 (12)	0.0625 (13)	0.0107 (9)	−0.0018 (10)	0.0021 (10)
C7	0.0533 (10)	0.0600 (10)	0.0498 (10)	0.0030 (8)	−0.0007 (8)	−0.0018 (8)
C8	0.0810 (13)	0.0845 (15)	0.0650 (13)	0.0261 (12)	0.0004 (12)	0.0052 (12)
C9	0.0862 (16)	0.0871 (16)	0.0849 (18)	0.0310 (13)	0.0016 (14)	−0.0089 (14)
C10	0.0905 (17)	0.0927 (17)	0.0752 (17)	0.0148 (14)	0.0108 (13)	−0.0277 (14)
C11	0.0877 (16)	0.0905 (17)	0.0504 (12)	0.0048 (13)	0.0065 (11)	−0.0092 (11)
C12	0.0598 (10)	0.0610 (11)	0.0476 (10)	−0.0026 (8)	0.0029 (8)	−0.0012 (8)
C13	0.124 (2)	0.0571 (13)	0.114 (2)	0.0010 (14)	0.0021 (19)	0.0205 (14)
C14	0.0516 (8)	0.0477 (8)	0.0540 (11)	−0.0075 (7)	−0.0029 (8)	−0.0012 (8)
C15	0.0549 (10)	0.0556 (10)	0.0731 (14)	0.0019 (7)	0.0007 (9)	0.0058 (10)
C16	0.0793 (13)	0.0602 (11)	0.0842 (15)	0.0131 (10)	−0.0135 (13)	−0.0024 (12)
C17	0.110 (2)	0.0502 (12)	0.0916 (19)	−0.0074 (12)	−0.0226 (15)	0.0083 (12)
C18	0.112 (2)	0.0664 (14)	0.116 (3)	−0.0274 (15)	0.0132 (18)	0.0180 (15)
C19	0.0705 (13)	0.0671 (14)	0.106 (2)	−0.0147 (10)	0.0195 (13)	0.0097 (13)
C20	0.0529 (9)	0.0382 (8)	0.0467 (9)	0.0036 (7)	0.0034 (7)	−0.0001 (7)
C21	0.0804 (16)	0.0569 (12)	0.0534 (13)	0.0099 (10)	0.0133 (10)	0.0009 (9)
C22	0.122 (2)	0.0656 (14)	0.0500 (12)	0.0125 (14)	−0.0011 (13)	−0.0161 (11)
C23	0.0982 (17)	0.0576 (12)	0.0752 (15)	−0.0024 (11)	−0.0260 (14)	−0.0107 (11)
C24	0.0624 (12)	0.0676 (13)	0.0733 (14)	−0.0109 (9)	−0.0138 (10)	−0.0027 (11)
C25	0.0527 (9)	0.0622 (11)	0.0536 (10)	−0.0032 (8)	0.0018 (8)	−0.0064 (8)

### Geometric parameters (Å, °)

**Table:**

P—O2	1.4675 (14)	C11—C12	1.410 (3)
P—O1	1.6138 (15)	C11—H11A	0.9300
P—C14	1.7835 (18)	C13—H13A	0.9600
P—C20	1.7948 (18)	C13—H13B	0.9600
O1—C1	1.392 (2)	C13—H13C	0.9600
N1—N2	1.335 (2)	C14—C15	1.380 (3)
N1—C7	1.357 (2)	C14—C19	1.391 (3)
N2—N3	1.337 (2)	C15—C16	1.385 (3)
N2—C2	1.427 (2)	C15—H15A	0.9300
N3—C12	1.351 (3)	C16—C17	1.377 (4)
C1—C2	1.382 (3)	C16—H16A	0.9300
C1—C6	1.387 (3)	C17—C18	1.362 (4)
C2—C3	1.392 (3)	C17—H17A	0.9300
C3—C4	1.385 (3)	C18—C19	1.379 (4)
C3—H3B	0.9300	C18—H18A	0.9300
C4—C5	1.371 (4)	C19—H19A	0.9300
C4—C13	1.515 (3)	C20—C25	1.392 (3)
C5—C6	1.385 (3)	C20—C21	1.393 (3)
C5—H5A	0.9300	C21—C22	1.392 (4)
C6—H6A	0.9300	C21—H21A	0.9300
C7—C12	1.394 (3)	C22—C23	1.359 (4)
C7—C8	1.420 (3)	C22—H22A	0.9300
C8—C9	1.365 (4)	C23—C24	1.376 (4)
C8—H8A	0.9300	C23—H23A	0.9300
C9—C10	1.407 (4)	C24—C25	1.381 (3)
C9—H9A	0.9300	C24—H24A	0.9300
C10—C11	1.357 (4)	C25—H25A	0.9300
C10—H10A	0.9300		
			
O2—P—O1	115.53 (10)	N3—C12—C7	108.62 (16)
O2—P—C14	113.48 (8)	N3—C12—C11	129.8 (2)
O1—P—C14	100.15 (8)	C7—C12—C11	121.60 (19)
O2—P—C20	112.37 (9)	C4—C13—H13A	109.5
O1—P—C20	105.04 (8)	C4—C13—H13B	109.5
C14—P—C20	109.30 (8)	H13A—C13—H13B	109.5
C1—O1—P	123.55 (12)	C4—C13—H13C	109.5
N2—N1—C7	102.17 (15)	H13A—C13—H13C	109.5
N1—N2—N3	116.91 (16)	H13B—C13—H13C	109.5
N1—N2—C2	123.51 (16)	C15—C14—C19	119.22 (18)
N3—N2—C2	119.59 (15)	C15—C14—P	123.30 (13)
N2—N3—C12	102.88 (15)	C19—C14—P	117.40 (16)
C2—C1—C6	119.13 (19)	C14—C15—C16	120.55 (19)
C2—C1—O1	118.37 (16)	C14—C15—H15A	119.7
C6—C1—O1	122.45 (18)	C16—C15—H15A	119.7
C1—C2—C3	120.14 (18)	C17—C16—C15	119.5 (2)
C1—C2—N2	121.79 (16)	C17—C16—H16A	120.3
C3—C2—N2	117.97 (18)	C15—C16—H16A	120.3
C4—C3—C2	121.0 (2)	C18—C17—C16	120.3 (2)
C4—C3—H3B	119.5	C18—C17—H17A	119.8
C2—C3—H3B	119.5	C16—C17—H17A	119.8
C5—C4—C3	118.1 (2)	C17—C18—C19	120.8 (2)
C5—C4—C13	120.9 (2)	C17—C18—H18A	119.6
C3—C4—C13	121.0 (3)	C19—C18—H18A	119.6
C4—C5—C6	122.0 (2)	C18—C19—C14	119.6 (2)
C4—C5—H5A	119.0	C18—C19—H19A	120.2
C6—C5—H5A	119.0	C14—C19—H19A	120.2
C5—C6—C1	119.7 (2)	C25—C20—C21	119.38 (18)
C5—C6—H6A	120.2	C25—C20—P	122.57 (13)
C1—C6—H6A	120.2	C21—C20—P	118.05 (15)
N1—C7—C12	109.42 (16)	C22—C21—C20	119.3 (2)
N1—C7—C8	129.50 (19)	C22—C21—H21A	120.3
C12—C7—C8	121.07 (18)	C20—C21—H21A	120.3
C9—C8—C7	116.0 (2)	C23—C22—C21	120.7 (2)
C9—C8—H8A	122.0	C23—C22—H22A	119.7
C7—C8—H8A	122.0	C21—C22—H22A	119.7
C8—C9—C10	122.6 (2)	C22—C23—C24	120.4 (2)
C8—C9—H9A	118.7	C22—C23—H23A	119.8
C10—C9—H9A	118.7	C24—C23—H23A	119.8
C11—C10—C9	122.2 (2)	C23—C24—C25	120.1 (2)
C11—C10—H10A	118.9	C23—C24—H24A	119.9
C9—C10—H10A	118.9	C25—C24—H24A	119.9
C10—C11—C12	116.5 (2)	C24—C25—C20	119.98 (19)
C10—C11—H11A	121.7	C24—C25—H25A	120.0
C12—C11—H11A	121.7	C20—C25—H25A	120.0
			
O2—P—O1—C1	69.76 (16)	N1—C7—C12—N3	0.0 (2)
C14—P—O1—C1	−167.96 (14)	C8—C7—C12—N3	179.2 (2)
C20—P—O1—C1	−54.64 (15)	N1—C7—C12—C11	179.55 (19)
C7—N1—N2—N3	−0.5 (2)	C8—C7—C12—C11	−1.2 (3)
C7—N1—N2—C2	179.47 (16)	C10—C11—C12—N3	−179.2 (2)
N1—N2—N3—C12	0.5 (2)	C10—C11—C12—C7	1.3 (3)
C2—N2—N3—C12	−179.50 (16)	O2—P—C14—C15	169.14 (18)
P—O1—C1—C2	145.53 (15)	O1—P—C14—C15	45.41 (19)
P—O1—C1—C6	−36.9 (2)	C20—P—C14—C15	−64.59 (19)
C6—C1—C2—C3	2.4 (3)	O2—P—C14—C19	−14.0 (2)
O1—C1—C2—C3	−179.93 (17)	O1—P—C14—C19	−137.76 (18)
C6—C1—C2—N2	−174.01 (18)	C20—P—C14—C19	112.24 (19)
O1—C1—C2—N2	3.7 (3)	C19—C14—C15—C16	−0.2 (3)
N1—N2—C2—C1	−47.2 (3)	P—C14—C15—C16	176.57 (17)
N3—N2—C2—C1	132.8 (2)	C14—C15—C16—C17	0.7 (4)
N1—N2—C2—C3	136.33 (19)	C15—C16—C17—C18	−1.6 (4)
N3—N2—C2—C3	−43.7 (2)	C16—C17—C18—C19	2.1 (5)
C1—C2—C3—C4	−0.9 (3)	C17—C18—C19—C14	−1.6 (5)
N2—C2—C3—C4	175.64 (18)	C15—C14—C19—C18	0.6 (4)
C2—C3—C4—C5	−1.0 (3)	P—C14—C19—C18	−176.3 (2)
C2—C3—C4—C13	−179.0 (2)	O2—P—C20—C25	−151.46 (15)
C3—C4—C5—C6	1.4 (4)	O1—P—C20—C25	−25.08 (17)
C13—C4—C5—C6	179.3 (3)	C14—P—C20—C25	81.63 (17)
C4—C5—C6—C1	0.1 (4)	O2—P—C20—C21	29.44 (18)
C2—C1—C6—C5	−2.0 (3)	O1—P—C20—C21	155.82 (14)
O1—C1—C6—C5	−179.60 (18)	C14—P—C20—C21	−97.47 (16)
N2—N1—C7—C12	0.33 (19)	C25—C20—C21—C22	1.0 (3)
N2—N1—C7—C8	−178.8 (2)	P—C20—C21—C22	−179.91 (17)
N1—C7—C8—C9	179.6 (2)	C20—C21—C22—C23	1.2 (3)
C12—C7—C8—C9	0.6 (3)	C21—C22—C23—C24	−1.4 (4)
C7—C8—C9—C10	0.0 (4)	C22—C23—C24—C25	−0.6 (4)
C8—C9—C10—C11	0.2 (5)	C23—C24—C25—C20	2.7 (3)
C9—C10—C11—C12	−0.8 (4)	C21—C20—C25—C24	−2.9 (3)
N2—N3—C12—C7	−0.3 (2)	P—C20—C25—C24	178.04 (16)
N2—N3—C12—C11	−179.8 (2)		
